# Drawing the Excalibur bug from the stone: adding credibility to the double-edged sword hypothesis of coreid evolution (Hemiptera, Coreidae)

**DOI:** 10.3897/zookeys.1043.67730

**Published:** 2021-06-14

**Authors:** Royce T. Cumming, Stéphane Le Tirant

**Affiliations:** 1 Montreal Insectarium, 4581 rue Sherbrooke est, Montréal , H1X 2B2, Québec, Canada Montreal Insectarium Montréal Canada; 2 Richard Gilder Graduate School, American Museum of Natural History, New York, NY 10024, USA American Museum of Natural History New York United States of America; 3 Biology, Graduate Center, City University of New York, NY, USA City University of New York New York United States of America

**Keywords:** Autotomy, Burmese, Cenomanian, Cretaceous, extinct, fossil, leaf-footed bugs, Mesozoic

## Abstract

A new genus and species of exaggerated antennae Coreidae is described from Myanmar amber of the Late Cretaceous (Cenomanian stage). *Ferriantennaexcalibur***gen. et sp. nov.** appears related to another Cretaceous coreid with exaggerated antennae, *Magnusantenna* Du & Chen, 2021, but can be differentiated by the fourth antennal segment which is short and paddle-like, the undulating shape of the pronotum and mesonotum, and the shorter and thicker legs. The new coreid, with elaborately formed antennae and simple hind legs instead of the typical extant coreid morphology with simple antennae and elaborately formed hind legs, begs the question: why were the elaborate features of the antennae lost in favor of ornate hind legs? Features that are large and showy are at higher risk of being attacked by predators or stuck in a poor molt and subjected to autotomy and are therefore lost at a higher rate than simple appendages. We hypothesize that because elaborate antennae play an additional significant sensory role compared to elaborate hind legs, that evolutionarily it is more costly to have elaborate antennae versus elaborate hind legs. Thus, through the millenia, as coreid evolution experimented with elaborate/ornate features, those on the antennae were likely selected against in favor of ornate hind legs.

## Introduction

**Figure 1.**
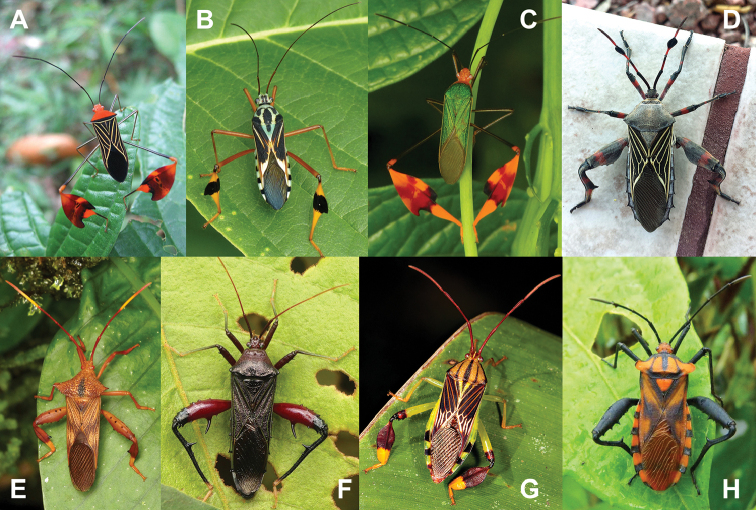
Examples of ornamentation in live extant coreids. Images **A–C, G** with expansions on the hind tibiae. Images **D–H** with hind leg spination **D** with expansions to the third antennal segment. Images **B, C, E–H** photographed by Andreas Kay (Ecuador), other images with appropriate citations given individually **A***Anisocelisflavolineata* from Veraguas Province, Panama, photographed by Dirk van der Made (Netherlands) **B** Unidentified Coreidae from Ecuador **C***Anisoscelisfoliacea* from Ecuador **D***Thasus* sp. from Santa Cruz County, Arizona, USA, photographed by Alan Schmierer (USA) **E** Unidentified Coreidae from Ecuador **F** Unidentified Coreidae from Ecuador **G***Meluchaquinquelineata* from Ecuador **H**Piezogastercf.humeralis from Ecuador.

The coreids (leaf-footed bugs) are a diverse group of hemipterans with a cosmopolitan distribution (of ~3100 species in ~260 genera; Henry 2017) and are known for their often-elaborate expansions and ornamentation of their hind femora and hind tibiae, and in some cases the humeral angles of the pronutum are even exaggerated (Fig. 1; Dolling 2006). These adaptations have been reported as being used for intraspecific competition/display (Eberhard 1998; Procter et al. 2012) and sensorial capabilities (for features on the antennae; Gonzaga-Segura et al. 2013). The superfamily Coreoidea as a whole has been recovered as monophyletic with an age of ~125 mya within recent fossil calibrated phylogenetic analyses (Johnson et al. 2018). Interestingly, while the Coreoidea has been recovered as monophyletic, the current internal taxonomic organizations have not (Forthman et al. 2019). When many of the morphological features which have been used in analyses in the past were reviewed alongside these recent large-scale molecular analyses it was found that most clades had few synapomorphies which define them, and most morphological features were found to be homoplastic (Forthman et al. 2019).

**Figure 2.**
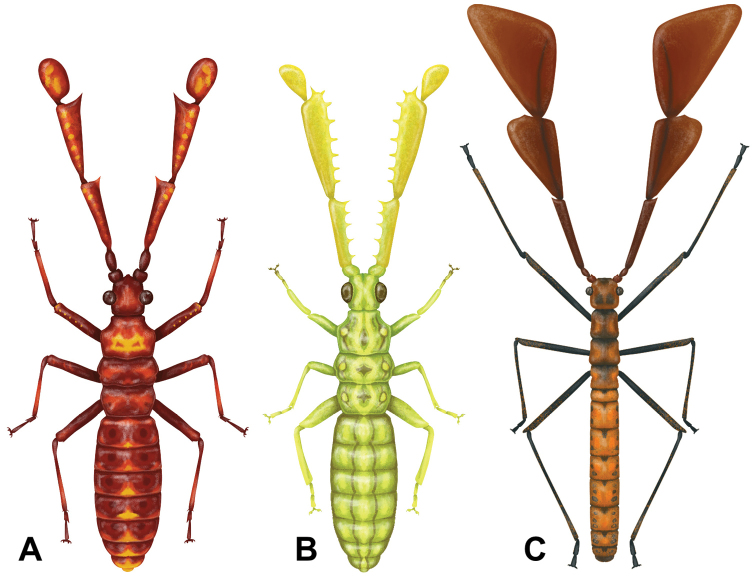
Artist recreation of the presently known three Cretaceous coreids with elaborate antennae. Illustrations by Liz Sisk (USA). Dorsal habitus scaled to same uniform length to highlight the antennae to body ratios. Colorations are artistic recreations based upon extant coreids rather than the actual specimen, whose color was not preserved in the amber **A***Ferriantennaexcalibur* gen. et sp. nov. **B***Ferriantenna* “club-like antennae” **C***Magnusantennawuae*.

The first reported Coreidae species from Cretaceous Burmese amber was the recently described *Magnusantennawuae* Du & Chen, 2021 (Fig. 2C), and was only the fifth species of coreid described from the Cretaceous (the others being impression fossils, not amber inclusions; Du et al. (2021)). Du et al. (2021) presented the first coreid from the Cretaceous with expansions on the antennae, a feature that is also seen in a few extant coreid species (Fig. 1D), but what was notable about their description was that the antennae were far more elaborate than any known extant species.

Unfortunately, with the fossil record of coreids rather fragmented and often from partial or nymphal specimens this still leaves a great deal of confusion surrounding their evolutionary history. Thankfully with nymphal antennae morphology rather stable into adulthood (Du et al. 2021) recent and herein described fossilized nymphs present a unique opportunity to understand the possible origin of elaborate morphological features.

## Materials and methods

The amber containing the holotype specimen was collected from the well-known Hukawng Valley in northern Myanmar, a prolific site of amber excavation (Grimaldi et al. 2002). The age of this amber deposit is estimated to be ~98.79 ± 0.62 million years old, within the Cenomanian stage of the Cretaceous (Shi et al. 2012). The holotype specimen described herein was morphologically reviewed using a 2x-225x trinocular boom stand stereo microscope (#ZM-4TW3-FOR-20MBI3) and photographs were taken with the attached high-speed 20MP camera (#MU2003-BI-CK) (AmScope, Irvine, USA). Illumination was from a 6-Watt LED dual gooseneck illuminator lit by an #85-265VAC/50-60Hz lighting unit (AmScope, Irvine, USA). Measurements were taken using AmLite digital camera software for Mac OS X 10.8 64-bit which was calibrated with a microscope stage calibration slide (#MR095), 0.01mm div. (AmScope, Irvine, USA). Adobe Photoshop Elements 13 (Adobe Inc., San Jose, USA) was used as post-processing software.

Illustrations were done by scientific illustrator Liz Sisk (Washington D.C., USA) using either photographs of the holotype, photographs saved from various online sources illustrating non-type specimens or recreated from the photographs/illustration presented in Du et al. (2021) in order to present side-by-side images of a uniform style.

The holotype specimen is deposited within the Montreal Insectarium, Montreal, Quebec, Canada (IMQC).

## Systematic paleontology

### Class Insecta Linnaeus, 1758

**Order Hemiptera Linnaeus, 1758**

**Family Coreidae Leach, 1815**

**Subfamily Coreinae Leach, 1815**

#### Genus Ferriantenna gen. nov.

Taxon classificationAnimaliaHemipteraCoreidae

155F95C1-0BDA-5C7D-9858-EED28EAF6E10

http://zoobank.org/40E251C3-987F-4F74-A89B-6545E487EDC8

##### Type species.

*Ferriantennaexcalibur* gen. et sp. nov., herein designated

##### Taxonomic remarks.

The taxonomic placement of this genus is rather uncertain, largely owing to the lack of adult specimens to allow review of genitalia, wing venation, and presence or lack of ocelli. Morphologically this genus appears to be closely related to *Magnusantenna* Du & Chen, 2021 based upon the elaborate antennae, square head shape, and long abdomen with parallel margins. Based upon this assumed close relationship we tentatively place this new genus and species within the Coreinae alongside *Magnusantenna* but would not be surprised if a taxonomic adjustment is necessary once adult specimens are hopefully one day recovered. Additional higher taxonomic possibilities, which can be ruled out, are Yuripopovinidae due to the lack of a distinct collar in our new taxon (Azar et al. 2011). Further, Yuripopovinidae typically have cylindrical antennomeres in cross section (although the recently described *Reticulatitergumhui*Du et al. 2019 does have a terminal antennomere which is flattened and rather similar in shape to our *Ferriantenna* gen. nov. (Fig. 2A; Du et al. 2019)). An additional clade which can have similar general habitus morphology are the Alydidae (particularly the Micrelytrinae which can have thin parallel-sided bodies and long legs very similar to *Magnusantennawuae*; Fig. 2C). The Alydidae can be differentiated from Coreidae by the length of the bucculae, with the bucculae shorter, not extending posteriorly beyond the base of the antennae in Alydidae but longer in Coreidae, extending posteriorly beyond the base of the antennae (Swanson 2011). Within *Magnusantennawuae*Du et al. (2021) clearly state that the bucculae are long extending posteriorly beyond the base of the antennae and therefore due to this feature would fit within Coreidae. Unfortunately, the amber piece that our *Ferriantenna* gen. nov. is within is too thick to clearly see the ventral surface of the head, but it does appear that the bucculae are longer than the base of the antennae and therefore more likely a Coreidae than an Alydidae.

The general morphological features of this genus fit well within Coreinae, namely the expanded antennal segments, the length ratios of the various antennomeres (the second and third segments of similar lengths), the smooth pronotum, and the straight femora and tibiae (Schuh and Slater 1995). At present there are three other subfamilies recognized within the coreids: Hydarinae, Meropachyinae, and Pseudophloeinae (CoreoideaSF Team 2021). The following features characterize each of the other subfamilies and help to add credibility to this genus being placed within Coreinae. Hydarinae have the third antennomere more than twice as long as the second (in *Magnusantenna* and *Ferriantenna* gen. nov. these segments are similar in length (Packauskas 1994)). The subfamily Pseudophloeinae is difficult to morphologically distinguish from other coreids as different authors consider different features significant for differentiation (e.g., Packauskas 1994; Moulet 1995; Schuh and Slater 1995; Hamouly et al. 2010; Schuh and Weirauch 2020). Due to the multiple morphological features which liken our genus to Coreinae we are fairly confident that these fossils do not fall within Pseudophloeinae. Meropachyinae are a small subfamily restricted to the western hemisphere and have a distal spine on the apex of the metatibiae and the metafemora are prominently thickened, notably broader than the pro- and mesofemora (Packauskas 1994; Brailovsky and Barrera 2009). Coreinae has repeatedly been recovered as paraphyletic with regards to Meropachyinae and based upon the typical Meropachyinae leg morphology we expect these fossil coreids do not fall within this clade but likely somewhere else within the Coreinae (Forthman et al. 2019, 2020; Kieran et al. 2019). Review of spermatheca within Coreidae by Pluot-Sigwalt and Moulet (2020) found that Hydarinae and Pseudophloeinae are morphologically unique but that Coreinae and Meropachyinae were similar, adding credibility to phylogenetic results which don’t recover Coreinae and Meropachyinae as unique (Kieran et al. 2019; Forthman et al. 2019, 2020).

Within the Coreinae there are several tribes which have an antennomere that is enlarged (e.g., Nematopini or Chariesterini with only the singular third antennomere flattened; Fig. 1D; CoreoideaSF Team 2021). This similarity alone does not warrant a tribal placement and the authors hope that eventually fossils of adult specimens are recovered to help determine a more accurate taxonomic placement as no extant tribe fits morphologically well.

##### Diagnosis.

Antennae four segmented, long, but not longer than the body (head, thorax, and abdomen). First antennal segment short and robust (slightly longer than wide or about equal in length and width; always shorter than head length); second and third segments ornamented and quite variable in form interspecifically (can be marked throughout with granulation, setation, or prominent tubercles with margins straight or with spination), each at least three times longer than wide, with the third segment slightly wider and longer than the second segment; and the fourth segment is only slightly longer than head length, flat, and paddle-like, lacking intricate features/expansions as present on the second and third segments. Head approximately as long as wide, compound eyes spherical and variable in their size (can be large, occupying most of the lateral margins, or narrower, restricted to the center third and strongly protruding), located on the center of each side of the head. Pronotum with a margin that expands to the posterior third then contracts slightly. Mesonotum gently expands to the midline and then gently contracts to the posterior. Metanotum with margins that can be parallel or slightly rounded. Abdomen slender, notably longer than wide, with parallel margins. Legs stout, not particularly long. Femora approximately two times as wide as the tibiae, but of similar lengths. Tarsi with two segments, bearing two claws.

##### Differentiation.

Several features differentiate the new genus from the assumed closely related genus *Magnusantenna* Du & Chen, 2021. First, the length ratios of the exaggerated antennal segments differ as *Magnusantenna* has the fourth segment approximately as long as, but notably broader than the third segment, versus *Ferriantenna* gen. nov. which has the fourth segment notably shorter than the third segment, appearing paddle-like. Additionally, the thickness and lengths of the legs differentiate these two genera as *Magnusantenna* has long thin legs (such as the hind legs which exceed the apex of the abdomen), versus *Ferriantenna* gen. nov. which has femora which are notably thicker than the tibiae, and specifically for the hind leg it appears that when fully outstretched they fall short or at most reaching the apex of the abdomen but do not exceed it. The thorax and abdomen of *Ferriantenna* gen. nov. are also notably broader than the head width versus *Magnusantenna* which has a very slender and long abdomen, thinner than the width of the quadrate head. Finally, the pro- and mesonotum differ slightly between these two genera as *Magnusantenna* has a pronotum which expands steadily from the anterior to the posterior and the mesonotum is parallel sided, versus *Ferriantenna* gen. nov. which has the pronotum expanding for the anterior two thirds then slightly contracting, and the mesonotum appears to expand to approximately the middle and then contract to the posterior.

##### Discussion.

Typically, Heteroptera have five instars, as in hemimetabolous insects which they resemble the adults in most morphological features. Our examined specimen which is the type species for this new genus appears to be a fourth instar nymph like was described within Du et al. (2021) based on the following characters they reference from Schuh and Slater (1995): posterior margins of the hind buds not reaching the anterior margin of the first abdominal tergite; ocelli absent; and tarsi two-segmented. As was noted within Du et al. (2021) amber typically does not preserve large inclusions well which is likely why all of these species are being observed as nymphs.

In addition to our herein described species, we have also seen images shared online of an additional species within *Ferriantenna* gen. nov. distinctly different from our *Ferriantennaexcalibur* gen. et sp. nov. This second, undescribed *Ferriantenna* species has similar characteristics of the thorax, abdomen, and legs, and the fourth antennomere which is notably smaller and paddle-like (Fig. 2B). This undescribed species however differs in that it has the second and third antennal segments heavily armored with prominent tubercles and granulation, making the antennae appear like a medieval two-handed iron spiked mace (Boeheim 1890) instead of blade-like as is seem in *Ferriantennaexcalibur* gen. et sp. nov. This second species, known only from photos shared online of a singular specimen, was being publicly offered for sale on eBay has since been sold. Unfortunately, the specimen could not be traced/examined and therefore we are unaware whether this specimen will end up in a museum collection for research or with a private collector.

The difference in leg lengths between *Magnusantenna* and *Ferriantenna* gen. nov. is likely due to the size of the antennae in relation to the body, as the *Ferriantenna* gen. nov. are notably less expanded and therefore require less leverage to maintain a stable footing, versus *Magnusantenna* which needed the longer legs to create a larger footprint to balance the massive antennae.

##### Etymology.

The generic name is derived from Latin prefix *ferri* (meaning weapon) and Latin *antenna* (meaning yardarm of a ship/sail yard which was the origin of the “feeler or horn” of an insect; https://www.etymonline.com/search?q=antenna). This genus epithet is referring to the weapon-like appearance of the antennae of these insects (Fig. 2A, B). Gender is neuter.

#### Ferriantenna excalibur

Taxon classificationAnimaliaHemipteraCoreidae

gen. et sp. nov.

A3294CD0-4337-5046-9CC6-345C9A4BE180

http://zoobank.org/D28929A-DF04-4038-BB44-B23DAE46BB82

Figures 2A , 3 , 4

##### Material examined.

**Figure 3.**
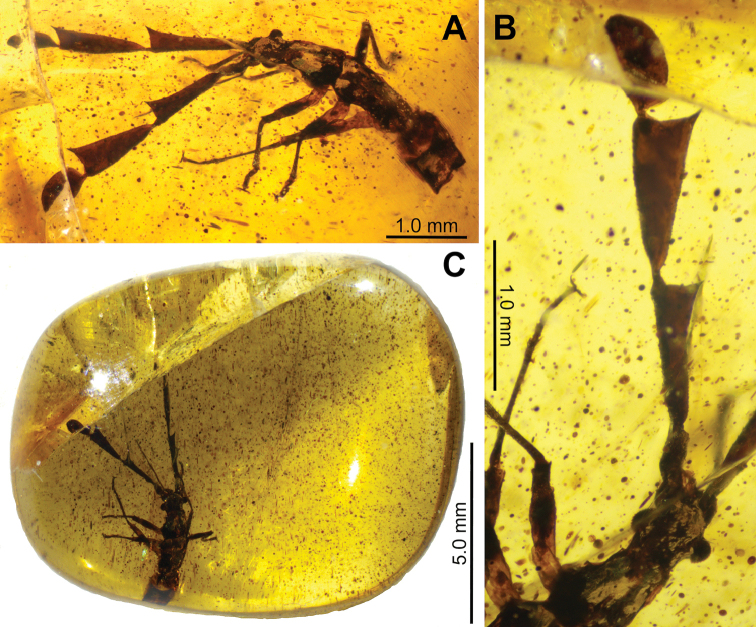
*Ferriantennaexcalibur* gen. et sp. nov. holotype **A** dorsolateral habitus **B** left antennae lateral and head dorsal **C** amber specimen #BHM10200800678 showing the inclusion.

***Holotype*:** Amber specimen #BHM10200800678. Flat and round rectangular piece of amber, approximately 1.0 cm by 1.1 cm with high clarity and small debris throughout that does not black visibility of the specimen (Fig. 3C). Specimen partially complete yet well-preserved, likely fourth instar. Missing the terminal two or three segments of the abdomen. Deposited in the Montreal Insectarium (IMQC). Unknown sex.

##### Type locality and horizon.

Kachin State, Myanmar; Upper Cretaceous ~98.79 ± 0.62 million years old (Shi et al. 2012). At present we are only aware of this genus and species being found in northern Myanmar from this stratum.

##### Differentiation.

At present this is the only formally described species within this new genus. Refer to the differentiation within the above genus section for discussion on the closely related *Magnusantennawuae*. We are aware of a second, undescribed *Ferriantenna* gen. nov. species (Fig. 2B) which differs by having the second and third antennomeres which are heavily armored with tubercles, not flattened with each segment narrow at the base and widening gradually to the sharply pointed anterior like is seen in *Ferriantennaexcalibur* gen. et sp. nov. (Fig. 2A). The elaborate antennae differentiate these extinct species from all known extant coreids which at most have a single slightly expanded antennal segment.

##### Description.

Mostly complete nymph which appears to be fourth instar. Sex unknown due to the instar stage and missing terminalia of the abdomen. Specimen complete except for the terminal two or three abdominal segments (Fig. 3A). Overall length (including antennae) 6.87 mm (measured to the end of the abdomen which is missing the terminal segments, so the actual length of the insect is slightly longer).

**Figure 4.**
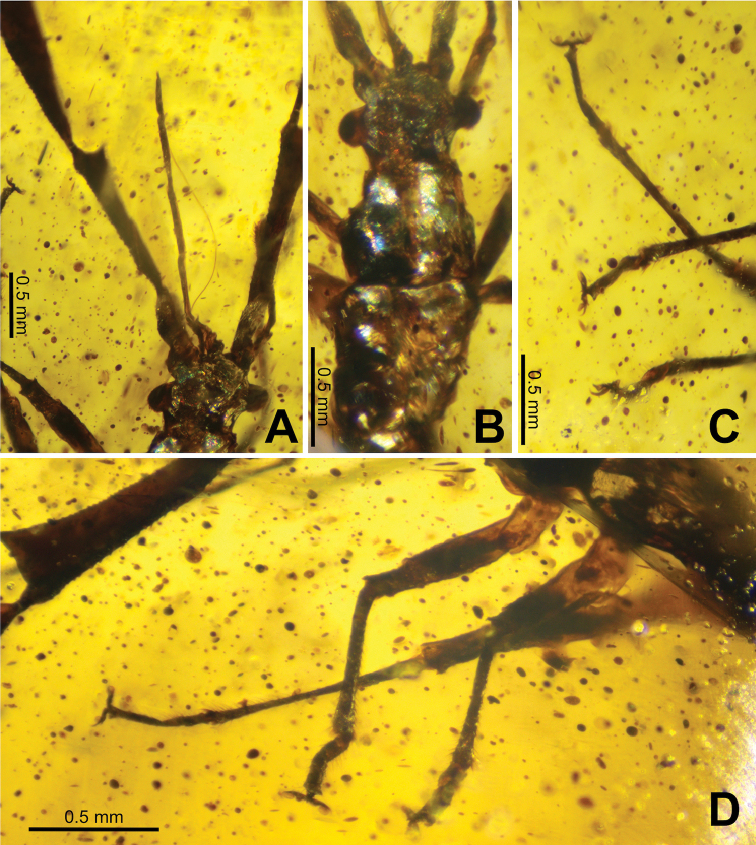
*Ferriantennaexcalibur* gen. et sp. nov. holotype **A** extended labium with stylet exposed to the right **B** head, pronotum, and mesonotum, dorsal **C** left tarsi and distal ends of the tibiae **D** legs on the left side, dorsolateral.

***Head*.** Antennal socket protruding from the front of the head (Fig. 4B), approximately 0.11 long by 0.20 mm wide, about as wide as the first antennomere. Head subquadrate, 0.50 mm long by 0.46 mm wide (without including compound eyes), including compound eyes head is 0.76 mm wide. Vertex relatively smooth, no notable textures or structures (Fig. 4B). Clypeus protruding slightly, labrum stout, not prominent. Labium tetramerous, fully extended reaches beyond the apex of the second antennomere, labiomeres one, two, and three similar in length, four approximately half as long as any of the others (Fig. 4A). Apex of the fourth labiomere sharply tapering to a fine point (Fig. 4A). Lengths: first labiomere 0.41 mm, second labiomere 0.51 mm, third labiomere 0.39 mm, fourth labiomere 0.26 mm. Compound eyes prominently protruding but not overly large, located in the center and taking up approximately one third of the lateral head margins (Fig. 4B).

***Antennae*.** Antennae tetramerous (Fig. 3B), length 3.22 mm, approximately equal in length to the damaged holotype body length (if the abdomen were complete the antennae would be slightly shorter in length than the body). First antennomere tubular, with sparse and short setae, 0.28 mm long and 0.14 mm wide. Antennomeres two through four appear to be laterally flattened due to the way the antennae are held in the amber. Second antennomere approximately right triangular in shape, with the anterior wide and the posterior narrow and the triangular expansion raised dorsally. Margins finely granular, with the dorsal margin marked with few fine setae, the ventral margin is marked with slightly longer and more prominent setae. Antennomere surfaces are relatively smooth, with minimal setae and only prominent granulation along the margins. Second antennomere length 1.08 mm and maximum width (on the anterior end) 0.39 mm. Third antennomere similar in shape and texture to the second antennomere but slightly wider throughout the length and on the anterior than the second antennomere; approximately right triangular in shape, with the anterior wide and the posterior narrow with the triangular expansion raised dorsally. Margins finely granular, with the dorsal margin with only fine setae, the ventral margin with slightly longer and more prominent setae. Antennomere surfaces relatively smooth, with minimal setae and fine granulation along the margins. Third antennomere 1.15 mm long and maximum width (on the anterior end) 0.52 mm. Fourth antennomere paddle-shaped and notably smaller than the previous two, with a narrow base expanding into a rounded segment; 0.71 mm long and 0.40 mm at the widest point (in the center). Fourth antennomere surfaces are more setose than the previous two antennomeres, marked throughout by moderate fine granulation. Margins with smaller and finer granulation and setae than on the previous two antennomeres.

***Thorax*.** Pronotum approximately an isosceles trapezium, anterior three fifths gradually expanding to the widest point, then the posterior two fifths converge slightly to the posterior (Fig. 4B). Dorsal surface of pronotum smooth, lacking prominent features. Overall pronotum length 0.73 mm, minimum width (on the anterior) 0.48 mm, width of the posterior 0.66 mm, maximum width on the posterior two fifths 0.72 mm. Mesonotum broader than long, with lateral margins expanding slightly on the anterior half and then contract slightly to the posterior (Fig. 4B). Overall mesonotum length 0.55 mm and greatest width 0.67 mm. Metanotum with anterior and posterior margins the same width, 0.60 mm, overall metanotum length 0.55 mm and maximum width (in the center) 0.65 mm.

***Legs*.** All legs of a similar morphology, only slight differences in length differentiate them (Fig. 4D). All femora of a uniform width, and all tibiae of a uniform width. Femora tubular, with a surface texture that is mostly smooth, but with a slight granular texture in places but not throughout. At the femora and tibiae joint the femora have a single spine-like projection on each side projecting outward and slightly towards the tibiae (Fig. 4D). Tibiae are half as wide as the femoral widths. Tibiae on the proximal end start out smooth but gradually become heavily setose along the ventral and lateral surfaces. At the apex of the tibiae the setae are rather prominent, and the setae continue on under the tarsomeres, albeit slightly more sparse, not as dense as the apex of the tibiae (Fig. 4C). Tarsi with two tarsomeres, apex with two distinct claws, each with a prominent pulvillus (Fig. 4C). Leg segment lengths: profemora 0.66 mm, mesofemora 0.60 mm, metafemora 0.77 mm, protibiae 0.62 mm, mesotibae 0.58 mm, metatibiae 0.94 mm.

***Abdomen*.** Abdomen notably damaged in the holotype. Disconnected from the body following the second segment, the remainder is mostly crushed, and the terminal two or three segments are missing (Fig. 3A). Greatest width approximately 0.55 mm. Abdomen without notable structures, margins parallel sided with rather smooth transitions from one segment to the next.

##### Etymology.

Noun in apposition, given for Excalibur, the mythical “sword in the stone” which was first described in the epic poem Merlin (about the mythical advisor to King Arthur), written by the French poet Robert de Boron sometime between 1195–1210 (Reeve and Wright 2007) which was a reworking of Geoffrey of Monmouth’s “Historia Regum Britanniae”, completed c. 1138 (Wright 1985). Within this poem is the first mention of Excalibur being the sword in the stone, which could only be removed by the true king of England. We felt that this specific epithet was fitting as this group of insects with exaggerated antennae were first described as a possible “double edged sword in evolution” as these elaborate antennae went extinct (Du et al. 2021). We felt this witty description, coupled with the insect being trapped in stone (amber) was fitting for such a long lost, and therefore mythical species.

## Conclusion

Our understanding of antennae diversity of the region and period is expanded with the description of this new genus and species of elaborately antennaed coreid from Cretaceous amber. These elaborate features have for the most part been lost from the antennae through the millennia and are now primarily found on the hind legs. Extant coreids primarily have the expansions on antennae restricted to a single antennal segment (Fig. 1D), and expansions are notably less elaborate than in extinct coreids (Fig. 2). There are several hypotheses we think might have led to this shift.

First, we feel the presence of elaborate structures on the hind legs versus the antennae is likely much more manageable for terrestrial movement due to a lower center of gravity (such as escape from predators of nymphs which cannot fly) and for flight in adults (as it is likely that such large/relatively heavy antennae would be difficult for flight/have significant wind resistance when on the anterior of the individual).

Also, it is most often large, elaborate appendages on insects that are reported as being lost at a higher rate to potential predators or to an imperfect molt than simple limbs/antennae (Maginnis 2008; Emberts et al. 2016). The large and elaborate antennae might have been more likely to be lost than the simple legs of extinct coreids. It is worth noting that in modern coreids the elaborate hind limbs have been reported as significant for sexual selection and overall mimesis, so just like their sensory significant antennae, their hind legs are impactful to overall fitness if lost (Eberhard 1998). Perhaps the elaborate antennae of this ancient coreid lineage were indeed a double-edged sword, as Du et al. (2021) hypothesized and discussed its costs/benefits. By losing these antennae it was evolutionarily prohibitively more costly due to the impact on the ability to find a mate via pheromone signaling, finding potential food sources, or oviposition sites (Elgar et al. 2018), thus leading to the extinction of this lineage.

Perhaps the selection pressures discussed above have acted against having elaborate (potentially likely to be lost) structures on the receptor valuable antennae, and instead the much more expendable hind legs have become the target for evolutionary experimentation for elaborate structures within the coreids. The extinct lace bug *Gyaclavatorkohlsi* Wappler, Guilbert, Wedmann, Labandeira, 2015 lends credibility to this idea of evolutionary experimentation leading to elaborate antennae, which are subsequently lost. This fossil Eocene lace bug has an expanded fourth antennomere, a feature previously unknown within Tingidae, which has not survived into extant species (Wappler et al. 2015).

An additional likely possibility/contributing factor is that this lineage of elaborate antennaed coreids fell victim to the Cretaceous–Paleogene (K–Pg) mass extinction which occurred approximately 66 million years ago and marked the end of the Cretaceous (Renne et al. 2013). This period of significant ecological disruption resulted in the extinction of a majority of species with estimates for extinction of marine life as high at ~75% (Jablonski 1994), extensive disruption to terrestrial plant communities (Wilf and Johnson 2004), and massive decline in diversity in terrestrial invertebrates (Wilf et al. 2006).

Typically, only one segment is expanded in extant coreids with the most elaborate antennae (Fig. 1D), but not to such a drastic degree as in the Cretaceous coreids discussed herein. Thus, whatever causes led to the elimination this elaborate antennaed coreid lineage, we are left with only these interesting fossils for speculation as to the function.

## Supplementary Material

XML Treatment for Ferriantenna

XML Treatment for Ferriantenna excalibur
